# PREDICTION OF FINANCIAL FRAUD RISKS AMONG OLDER ADULTS: THE APPLICATION OF GA-XGBOOST MODEL

**DOI:** 10.1093/geroni/igae098.1396

**Published:** 2024-12-31

**Authors:** Jin Zhao, Yuanyuan Li, Hao Jin, Xuemin Zhang

**Affiliations:** Hebei University of Technology, TianJin, Tianjin, China (People’s Republic); Hebei University of Technology, TianJin, Tianjin, China (People’s Republic); Hebei University of Technology, TianJin, Tianjin, China (People’s Republic); Hebei University of Technology, TianJin, Tianjin, China (People’s Republic)

## Abstract

The importance of crime prevention can be more profound than correction efforts after crime occurs, highlighting the necessity to develop an early risk warning model that can accurately identify financial fraud risks among older adults. Utilizing data from the fourth Urban and Rural Survey on the Living Conditions of Older Adults conducted in 2015 in China, this study leverages a genetic algorithm (GA) to optimize the parameters of the XGBoost model, resulting in the creation of a GA-XGBoost-based financial fraud risk prediction model tailored for older adults. The efficiency of this novel model is evaluated against established machine learning algorithms, including LightGBM, Random Forest, Decision Tree, and SVM. Furthermore, SHAP values are employed for a detailed visual analysis of the factors most influential in predicting and understanding financial fraud risks among this demographic. We found that variables such as lower education level, older age, and loneliness increased risks for financial fraud. In contrast, increased awareness of rights protection and better sleep quality appear to have protective effects. A comparative assessment at both domestic and international levels, with references to the United States, Germany, and South Korea, highlights psychological vulnerability, cognitive functioning, age, and loneliness as financial fraud risks among older adults. This study offers recommendations for mitigating financial fraud risks from three angles: enhancing public awareness and education; implementing effective regulatory frameworks; and strengthening intergenerational connections. These suggestions will help lay the groundwork for more robust protective measures against financial fraud targeting older adults.

